# Fabrication of Nanoscale Pits with High Throughput on Polymer Thin Film Using AFM Tip-Based Dynamic Plowing Lithography

**DOI:** 10.1186/s11671-017-2319-y

**Published:** 2017-09-22

**Authors:** Yang He, Yanquan Geng, Yongda Yan, Xichun Luo

**Affiliations:** 1 The State Key Laboratory of Robotics and Systems, Robotics Institute, Harbin Institute of Technology, Harbin, Heilongjiang 150080 People’s Republic of China; 2 Center for Precision Engineering, Harbin Institute of Technology, Harbin, Heilongjiang 150001 People’s Republic of China; 3 Centre for Precision Manufacturing, Department of Design, Manufacture and Engineering Management, University of Strathclyde, Glasgow, UK

**Keywords:** AFM, PMMA film, Nanoscale pit, Nanogroove, Fast-scan nanolithography, DPL

## Abstract

We show that an atomic force microscope (AFM) tip-based dynamic plowing lithography (DPL) approach can be used to fabricate nanoscale pits with high throughput. The method relies on scratching with a relatively large speed over a sample surface in tapping mode, which is responsible for the separation distance of adjacent pits. Scratching tests are carried out on a poly(methyl methacrylate) (PMMA) thin film using a diamond-like carbon coating tip. Results show that 100 μm/s is the critical value of the scratching speed. When the scratching speed is greater than 100 μm/s, pit structures can be generated. In contrast, nanogrooves can be formed with speeds less than the critical value. Because of the difficulty of breaking the molecular chain of glass-state polymer with an applied high-frequency load and low-energy dissipation in one interaction of the tip and the sample, one pit requires 65–80 penetrations to be achieved. Subsequently, the forming process of the pit is analyzed in detail, including three phases: elastic deformation, plastic deformation, and climbing over the pile-up. In particular, 4800–5800 pits can be obtained in 1 s using this proposed method. Both experiments and theoretical analysis are presented that fully determine the potential of this proposed method to fabricate pits efficiently.

## Background

The recent and rapid development of nanotechnology has attracted increasing attention to the application of nanostructures in various fields, such as nanoelectromechanical systems, nanosensors, and nanophotonics. In particular, nanodots, defined as one-dimensional nanostructures, are widely utilized in the fields of high-density storage and preparation of quantum dots [1]. However, efficient fabrication of nanodots still faces enormous challenges. Many scholars have proposed various methods to fabricate nanodots on a wide variety of materials. Among them, the chemical synthesis method is widely used to obtain nanodots for most property detection and nanoscale devices [2]. However, it is difficult to determine the dimensions and spatial distribution of the nanodots using this method. These results in more effort required for location and manipulation in subsequent processes. Thus, many scholars have devoted resources to exploring more controllable methods to obtain nanodot structures with dimensions of several nanometers, such as focused ion beam lithography [3], electron beam lithography [4], and nanoimprint lithography [5]. However, the complexity, strict environmental requirements, and/or high cost greatly impede the applications of these techniques.

Since the atomic force microscope (AFM) was invented in 1986, it has been commonly utilized as a high-precision surface profiler [6]. When the interaction force between the AFM tip and the sample is enlarged to a relatively large value, such as several hundred nanonewtons or even several hundred micronewtons, the sample material can be removed by the sharp tip plastically, similar to a small cutting tool [7]. Chemical and thermal energies have also been introduced in the AFM system through local oxidation [8] or heating sample [9] to assist the removal of sample materials. It, therefore, results in some new manufacturing methods to extend the scope of the existing AFM tip-based nanolithography (TBN) methods. Among all of the TBN methods, the mechanical removal approach is the easiest and most flexible [10]. This method consists first of indentation and subsequent scratching actions on various materials, in which the tip-material interaction is strongly dependent on the type of material, such as metals [11], semiconductors [12, 13], and polymers [14]. By precisely controlling the tip-material interaction on the nanoscale, complex and high-precision nanostructures, such as nanodots, nanogrooves, and even 3D nanostructures, have been successfully fabricated. In particular, some scholars have performed AFM tip-based nanoindentation processes on the surface of semiconductor materials to obtain nanodot structures [15, 16]. In their studies, the crystalline defects caused by nanoindentation have been determined to be nucleation sites for InAs nanostructures. However, the relatively large hardness of the semiconductor materials could result in serious tip wear. Thus, some researchers have proposed carrying out the nanoindentation process on softer materials, such as polymer thin-film resist, to first fabricate nanodot structures. These nanodot structures could then be transferred to semiconductor materials by reactive ion etching (RIE) or wet etching processes [17]. Because of its low hardness and ultra-thin thickness, the resist layer could be penetrated with a relatively small normal load. Some scholars have proposed a two-step scratching approach to obtain nanodot arrays on a polycarbonate surface [18]. This method relies on the ripples of the materials formed by the AFM tip-based force constant scratching process. However, the spacing distance between adjacent nanodots only depends on the geometry of the AFM tip, and the formation mechanism of the nanodots remains unclear.

On the other hand, low throughput is a critical factor impeding the development of AFM tip-based nanofabrication methods. It has been demonstrated that the AFM tip-based nanoindentation process is time-consuming for obtaining large-scale nanodot structures [19]. To solve this problem, Vettiger et al. presented the concept of “Millipede,” which employs large arrays of micro-cantilevers operating in parallel to achieve ultra-high-density machining capability [20]. Considering the serious tip wear after a large-area scratching process, some scholars have proposed a novel intermittent-contact mode operation to reduce the tip-sample interaction force, thus decreasing the tip wear [21, 22]. However, the large arrays of micro-cantilevers used in this approach need complicated design and production processes, and a tedious process is required for adjusting the position of all tips on one probe to guarantee contact with the sample. Therefore, some researchers have modified the commercial AFM system, including hardware and software, to promote the high-speed machining capability [23–25]. In these methods, tips with one cantilever were employed. However, only nanogrooves can be fabricated using these approaches efficiently, and scratching with large speeds could also lead to serious tip wear. In addition to static processing with an AFM tip, AFM tip-based dynamic plowing lithography (DPL) has also attracted more and more attention recently; this process is conducted with the tapping mode of the AFM system. When increasing the drive amplitude of the cantilever, the AFM tip can penetrate the sample surface to achieve the machining process [26–28]. Because of the intermittent contact between the tip and sample in the DPL approach, the tip wear could be reduced, similar to the methods proposed in Refs. [21, 22]. The machined depth obtained by the DPL method is usually on the order of a few nanometers, which is suitable for fabricating nanostructures on a thin film, such as polymer thin-film resist and two-dimensional materials [29]. Moreover, in the DPL method, the cantilever of the AFM tip could be driven to oscillate at several thousands of hertz, which would result in the tip interacting with the sample surface many times in a short period. Thus, the DPL method could be a potential approach to fabricate nanoscale pit structures on a thin-film sample surface efficiently.

**Fig. 1:**
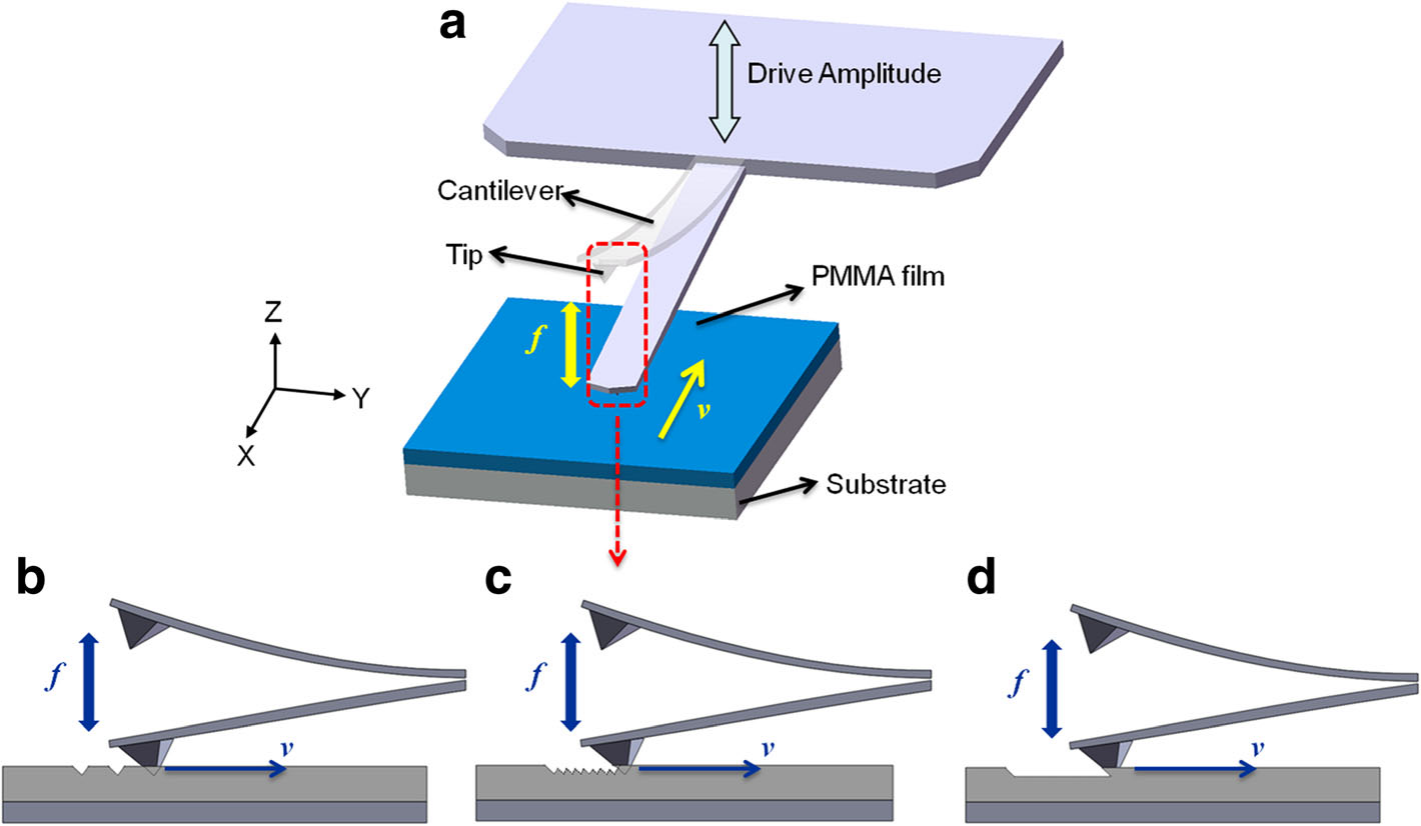
**a** Schematic of the line scratching process on PMMA film surface with the FSN method. The cantilever is oscillating at its resonance frequency *f* in the vertical direction. The scratching velocity *v* is modified along the fast scan direction. Various scratching velocity ranges are depicted: **b** high scratching velocity, **c** medium scratching velocity, and **d** low scratching velocity

In this study, a fast-scan nanolithography (FSN) method is presented based on the DPL fabrication approach and employing a commercial AFM system. Figure 1a shows a schematic of the nano-scratching process with a diamond-like carbon coating tip, which illustrates a view of the tip in contact with a poly(methyl methacrylate) (PMMA) thin film on a silicon substrate. The cantilever is driven near its resonance frequency to generate intermittent contact between the tip and the sample surface. The drive amplitude of the tip is sustained at a constant value by the control system (AM-AFM). The Nanoman module equipped on the AFM system is used for all machining process, and the scratching direction is chosen as parallel to the long axis of the cantilever. The effect of the scratching velocity on the machined nanostructures is studied. Moreover, the formation mechanism of the pit structure is also investigated.

## Methods

The concentration of the PMMA solution is 1.25 wt%, prepared by dissolving the PMMA powder with molecular weight Mw = 120,000 into chlorobenzene. The PMMA films are prepared by spinning the solution on a piece of single-crystal Si substrate, which is cleaned by successive ultrasonic baths in acetone and alcohol for approximately 10 min. The spinning speed is chosen to be 6000 rpm in the experiments to generate films with thicknesses of several tens of nanometers. Following the coating process, PMMA films are post-baked at 125 °C, which is near the glass transition temperature of PMMA, for 30 min.

The experiments are operated with a commercial AFM (Dimension Icon; Bruker Corporation, USA). A silicon tip is selected with a nominal spring constant of 42 N/m and resonant frequency of 320 kHz, provided by the manufacturer (TESPD; Bruker Corporation, USA). The tip side of the cantilever is hardened with a diamond-like carbon (DLC) coating for extended tip life. The measurement of nanostructures on the PMMA surface is set to tapping mode with a scan rate of 1 Hz and a scan line of 256. The cantilever system has to be tuned when using different probe. The setting point is tuned around 300 mV in this study. The images are processed by first-order flattening using the Nanoscope Analysis software provided by the Bruker Company.

The equipped Nanoman module in the AFM system is utilized for the scratching process in this study, which is widely adopted to design the trajectory of the tip to achieve desirable structures, like rectangles or circles, on the sample surface. To modify the surface, the drive amplitude value of the tip should be increased to *V*_w_ (writing), where the interaction between the tip and the PMMA film is promoted to guarantee the tip penetrates the sample surface. After the scratching process, the drive amplitude value of the tip is immediately plunged to *V*_r_ (reading) without changing the cantilever. Compared with the static plowing lithography method, the tip wear is proved to be very small with DPL, and thus, it can be neglected. By avoiding changing the probe and searching for the location of the nanostructure, this in situ imaging method can improve the efficiency of the scratching process. All experiments are operated at room temperature.

## Results and Discussion

Considering the speed limitation of the AFM PZT, scratching velocities in the range from 0.1 to 1000 μm/s are selected in the experimental tests. Figure 1 shows a schematic of the nanomachining process, including three velocity ranges. When scratching at a relatively large velocity (around several hundred micrometers per second), separate pits can be formed, as shown in Fig. 1b. When the scratching speed slows down to a medium value (around 100 μm/s), the pits can overlap with each other, as shown in Fig. 1c. As shown in Fig. 1d, when the scratching velocity reaches a relatively small value (dozens of micrometers per second), the pits can be transformed into nanogrooves. This result indicates that the distance between the two fabricated pits is dependent on the scratching velocity, which has a large influence on the fabricated nanopatterns.

**Fig. 2:**
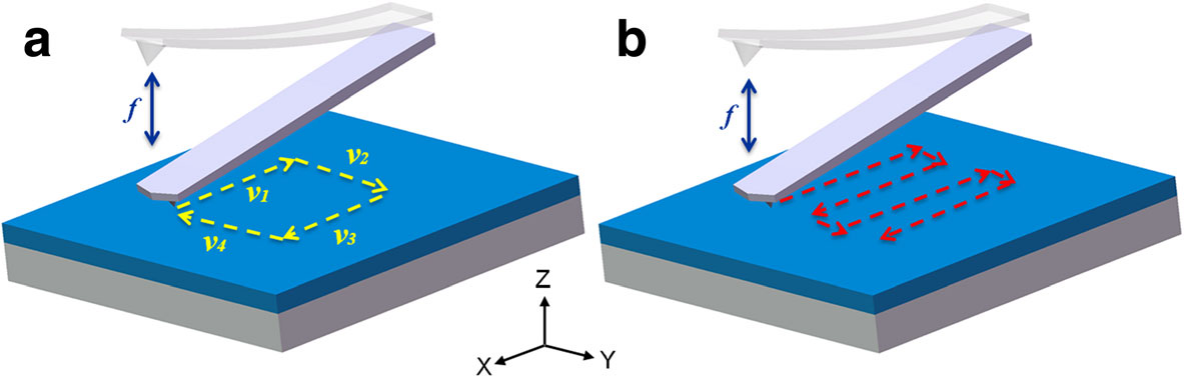
**a** Four typical scratching directions (*V*_1_, *V*_2_, *V*_3_, and *V*_4_) are chosen for nanomachining fabrication in this study. **b** The tip trajectory with the feed for large-area patterns

**Fig. 3:**
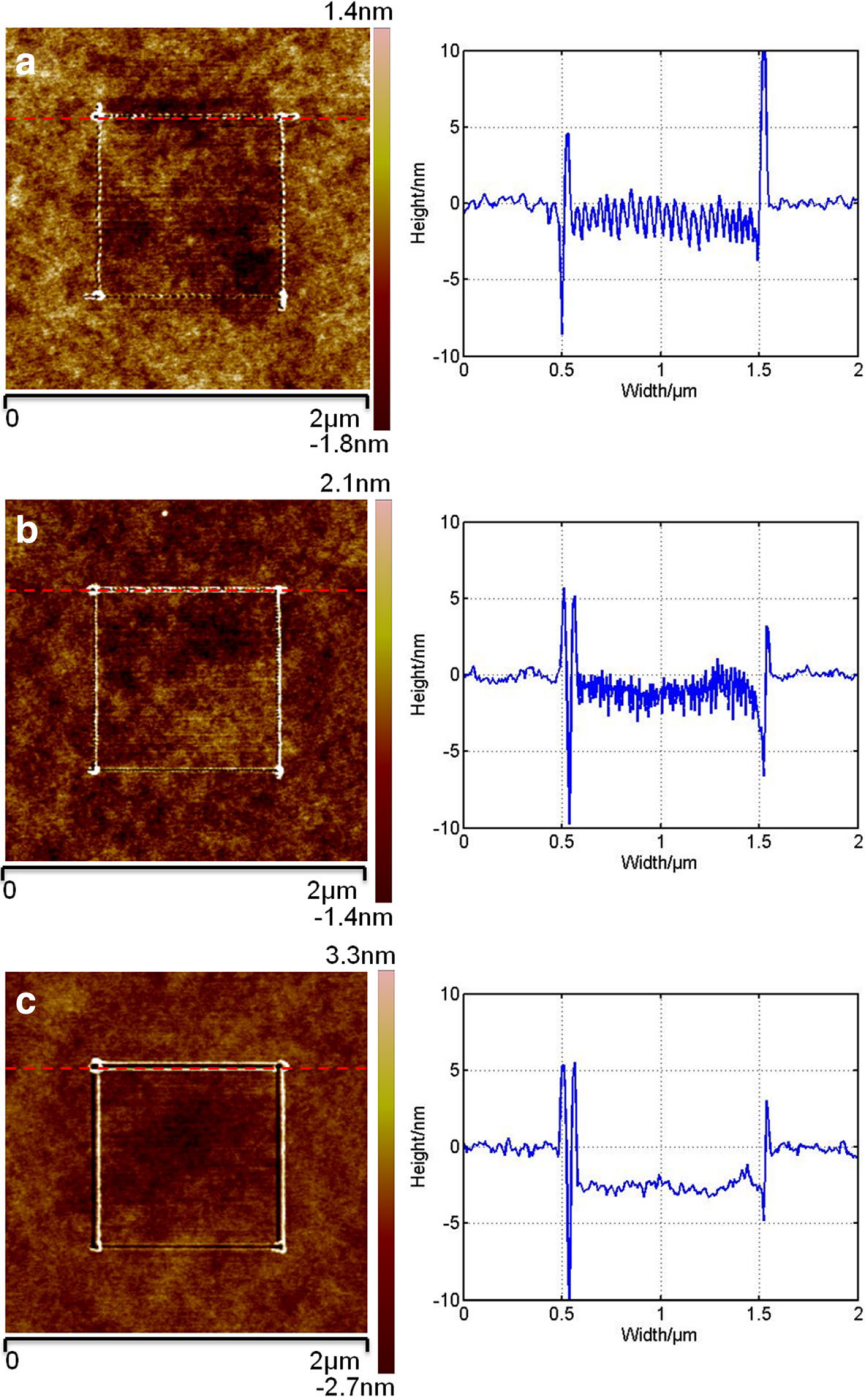
AFM images of three kinds of nanostructures and their cross-section with scratching velocities of **a** 200 μm/s, **b** 100 μm/s, and **c** 50 μm/s

In this study, four typical scratching directions are chosen, as shown in Fig. 2a. *V*_1_ and *V*_3_ represent scratching along the long axis of the cantilever; *V*_2_ and *V*_4_ are defined as scratching perpendicular to the long axis of the cantilever. The tip trajectories are obtained by controlling the AFM PZT. Figure 3 shows AFM images of square line nanostructures fabricated with different scratching velocities and the corresponding cross-sections of the nanostructures scratched with the direction *V*_1_, when the resonance frequency of the cantilever is 380 kHz. With a relatively large scratching velocity of 200 μm/s, continuous pits can be formed, as shown in Fig. 3a. For four scratching directions set in advance, pits would be formed immediately far less than 1 s, even though the scratching velocity slides away at the turning point of the two directions. With a medium scratching velocity (100 μm/s), no obvious pit can be found along the machining path and fluctuant nanostructures are formed, as shown in Fig. 3b. Only one pit with a much larger depth can be observed at the intersection of the two adjacent scratching paths, which can be explained as follows. During the period of the transformation between the two adjacent scratching paths, the scratching velocity should slow down to 0 and the tip can press into the sample surface more times than in the case of scratching, which may be the possible reason for generating a larger depth of pit. The scratching velocity of 100 μm/s can be considered a critical value for fabricating continuous pits on a PMMA thin film. Figure 3c shows the machined nanogrooves with a scratching velocity of 50 μm/s. From the cross-section of the machined nanogroove, it can be observed that the bottom of the nanogroove is relatively flat and an obvious depth of the nanogroove can be formed. Moreover, as shown in Fig. 3, because the tip plows on the PMMA film, there are no chips formed during the scratch and only pile-ups can be formed, on one side or both sides of the groove. In addition, the profiles of different sides of the obtained nanostructures are inconsistent when scratching with different directions, which is similar to the results using static lithography with an asymmetric tip. For other resist materials such as SU-8 or polystyrene (PS), the threshold values of the scratching velocity will be different from the one of PMMA film, owing to different stress relaxation modulus. However, their threshold values can be obtained through the scratching experiment by following the same approach of this study.

**Fig. 4:**
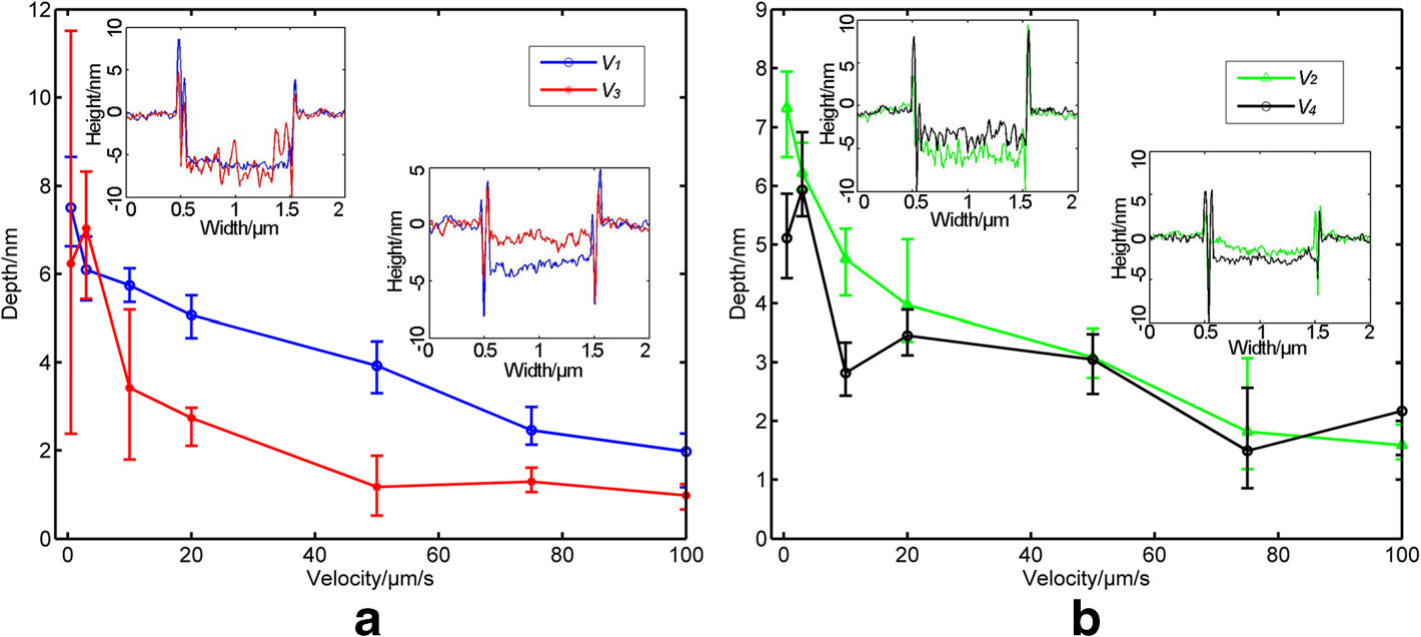
Dependence of groove depth on the scratching velocity in typical scratching directions: **a** *V*_1_ and *V*_3_, parallel to the long axis of the cantilever; **b** *V*_2_ and *V*_4_, perpendicular to the long axis of the cantilever. The insets show the cross-section of nanogrooves for scratching velocities of 0.5 and 50 μm/s

With scratching velocities less than 100 μm/s, nanogrooves with good quality can be obtained. Figure 4 shows the relationship between the machined depth of the nanogroove and the scratching velocity with the different scratching directions shown in Fig. 2a. For each nanogroove, the experimental depth is calculated by the average of five depth values at five different locations. The scratching distances for all scratching directions are the same—1 μm in this study. It can be observed that the machined depth decreases with increasing scratching velocity for all scratching directions. One possible reason can be explained as follows. For a scratching distance of 1 μm as selected in this study, the numbers of press operations under scratching velocities of 100 μm/s and 1 μm/s will be 3870 and 387,000, respectively. For the same scratching distance, a large number of press operations by the AFM tip can lead to a relatively large percentage of overlap between the adjacent press operations, which can result in a larger machined depth of the nanogroove. Furthermore, as shown in Fig. 4, the depths of the nanogrooves scratched in all directions are consistent when the scratching velocity is less than 5 μm/s, while the depth of the nanogroove machined in direction *V*_3_ becomes much smaller than the machined depths obtained by other directions with scratching velocities larger than 5 μm/s. Moreover, the error bars of the machined depths obtained in direction *V*_3_ are much larger when the scratching velocity is less than 5 μm/s than for others. One possible reason can be explained as follows. The geometric AFM probe used in this study is unsymmetrical, and a tilt of the probe caused by the typical cantilever slope of 12°, used to ensure that only the AFM tip will touch the sample surface, can result in a difference of the contact area between the tip and the sample surface with different scratching directions. For a scratching velocity of less than 5 μm/s, the overlapping area of the adjacent press operations is very large. Thus, the contact area between the tip and the sample surface is also extremely large. The influence of the scratching direction on the machined depth can therefore be negligible. However, the pile-up formed along the tip surface is also dependent on the scratching direction, which is similar to the static scratching process. Therefore, the pile-up cannot be formed steadily in the *V*_3_ scratching direction. The inserted figures in Fig. 4a, b are the cross-sections of typical nanogrooves machined with scratching velocities of 0.5 and 50 μm/s, respectively. From the cross-section of the nanogroove machined with a scratching velocity of 0.5 μm/s, the bottom of the nanogroove is fluctuant when scratching in the *V*_3_ direction, which can result in a relative large error bar for the machined depth. For scratching with a velocity of larger than 5 μm/s, the overlapping area with adjacent press operations becomes small. Thus, the scratching direction plays an important role, which can result in a relatively small machined depth obtained in the *V*_3_ scratching direction.

A PMMA thin film is a kind of time-dependent and viscoelastic material. Thus, the periodic load applied by the AFM tip may have an influence on the Young’s modulus of the sample. The general representation for the stress relaxation modulus *G* is defined by *G*_1_ and *G*_2_ [30]:

1 $$ G\left(\omega \right)={G}_1\left(\omega \right)+{iG}_2\left(\omega \right) $$

2 $$ {G}_1\left(\omega \right)=\left[{G}_r\right]+{\int}_{-\infty}^{+\infty}\frac{H\left(\tau \right){\omega}^2{\tau}^2}{1+{\omega}^2{\tau}^2}d\left(\ln \tau \right) $$

3 $$ {G}_2\left(\omega \right)={\int}_{-\infty}^{+\infty}\frac{H\left(\tau \right)\omega \tau}{1+{\omega}^2{\tau}^2}d\left(\ln \tau \right) $$

where *G*_r_ is a constant and *ω* is related to the frequency. *H*(*τ*) is the relaxation-time spectrum contributed to the stress relaxation, which has a relationship with the relaxation times between ln*τ* and ln*τ* + *d*(ln*τ*). When the excitation frequency is set to a value near the resonance frequency of the cantilever, which is 387 kHz, the modulus can reach a high value. From the calculation using the equations mentioned above, the PMMA thin film presents as glass state with an applied high-frequency load [30]. Because the tapping mode is used in the whole machining process, the interaction force and the energy dissipation between the AFM tip and the sample surface during the scratching process are relatively small, and even the drive amplitude *V*_w_/*V*_r_ is set to a relatively high value, ranging from 10 to 20. With these machining conditions, because of the glass-like property of the PMMA thin film and a relatively small applied load by the AFM tip, the chains between the polymer molecules cannot be broken and plastic deformation is difficult to be generated to modify the sample surface by one cycle of the press operation. However, the tip has sufficient energy (> 1~2 eV) to accomplish this during the first 20–30 times of press operation [27]. Thus, the chain bonds between the polymer molecules can be cut off to generate plastic deformation on the thin-film surface.

The spacing distance between the adjacent press operations is a critical parameter that has a relationship with the scratching velocity and the oscillating frequency of the tip. The distance of one pit line (*L*) can be obtained by the time used for one pit line (*t*) multiplied by the scratching velocity (*v*). The total number of AFM tip oscillations in one pit line (*N*) can be calculated using the oscillating frequency of the cantilever (*f*) multiplied by the time (*t*). Thus, the spacing distance between adjacent press operations (*D*) can be obtained by Eq. 4.

4 $$ D=\frac{L}{N}=\frac{v}{f} $$

**Fig. 5:**
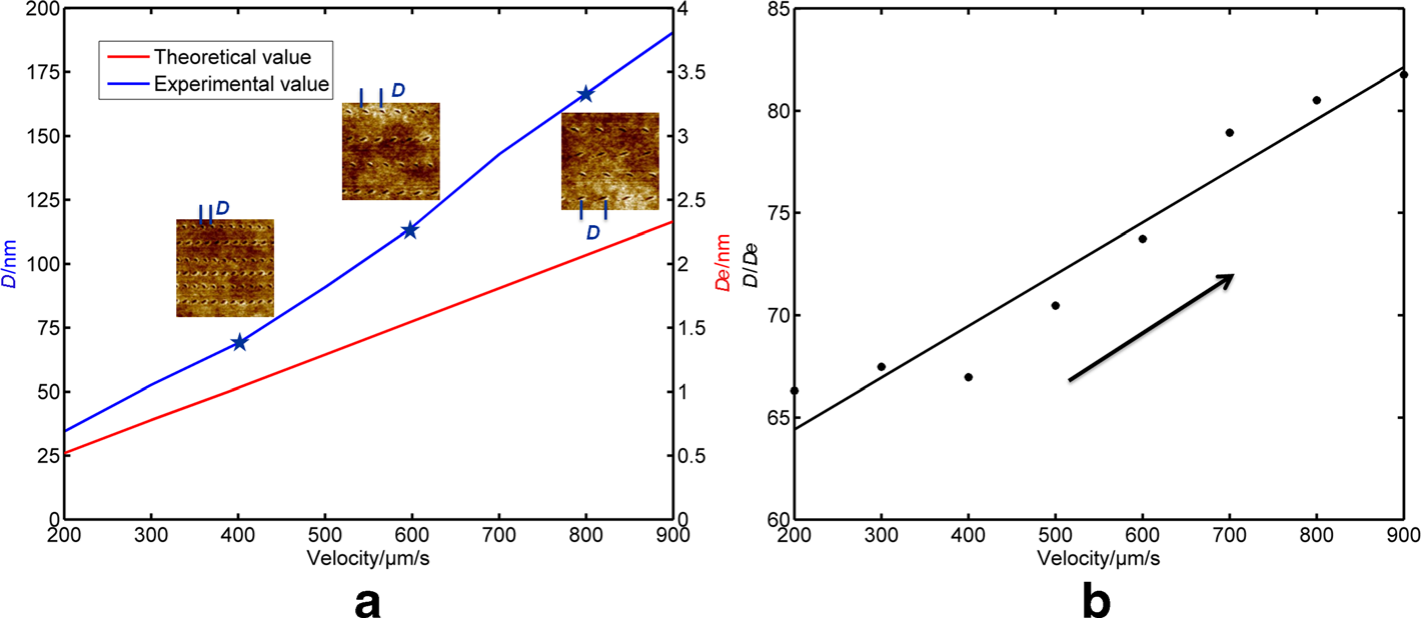
**a** Variation of *D* and *D*_e_ with scratching velocity (200–900 μm/s); insets show fabrication results for various scratching velocities. **b** Ratio of *D* to *D*_e_

The natural vibration frequency of the cantilever selected in this study is approximately 387 kHz. The drive frequency of the AFM system is chosen to be close to this value. As mentioned above, the scratching velocity should be selected in the range from 200 to 900 μm/s to guarantee formation of the pits. Thus, the spacing distance between the adjacent press operations (*D*_e_) during the scratching process can be calculated in the range from 0.52 to 2.33 nm, which is denoted as the red curve in Fig. 5a. The blue curve in Fig. 5a represents the relationship between the spacing distance between adjacent pits (*D*) obtained from the experiments and the scratching velocity. The inset AFM images are obtained for pits machined with three typical scratching velocities of 400, 600, and 800 μm/s. Therefore, the numbers of press operations for one pit formation can be calculated as the ratio of *D* to *D*_e_ shown in Fig. 5b. Assuming that the scratching velocity is a constant value, 4800–5800 pits can be generated on a PMMA thin film in 1 s, as calculated from the scratching length (*L*) and the spacing distance (*D*). From Fig. 5b, it can be observed that the number of press operations for one pit formation increases with increasing scratching velocity and are mostly in the range from 65 to 80. Considering the level terrain between the two pits is almost equal to the dimensions of pits, only approximately 32–40 press operations are required to break the polymer chains to generate plastic deformation of the sample surface, which is consistent with Cappella’s conclusion [27]. In addition, it can be concluded that it is easier to break the polymer chains when scratching with a relatively small velocity. In this study, the spring constant of the cantilever is identical. A stiffer cantilever could be used for the fabrication of pits, which results in a larger applied force and a higher resonance frequency. If a larger force is applied to the sample surface, the energy input is increased in each cycle. More energy dissipation is thus contributed to the deformation of the PMMA film. One pit can therefore be generated with decreased cycles. However, if the resonance frequency is increased for the oscillation system, the cycle of the press operation between the sample surface and the tip is thus increased. In addition, the energy dissipation would be increased in one cycle, owing to the setting point decreased in the experiments. The critical velocity may be determined by the value of the setting point. Based on the discussions above, the threshold value of the speed could be influenced by the applied force, the resonance frequency of the cantilever system, and the setting point, which will be the focus of future investigations.

**Fig. 6:**
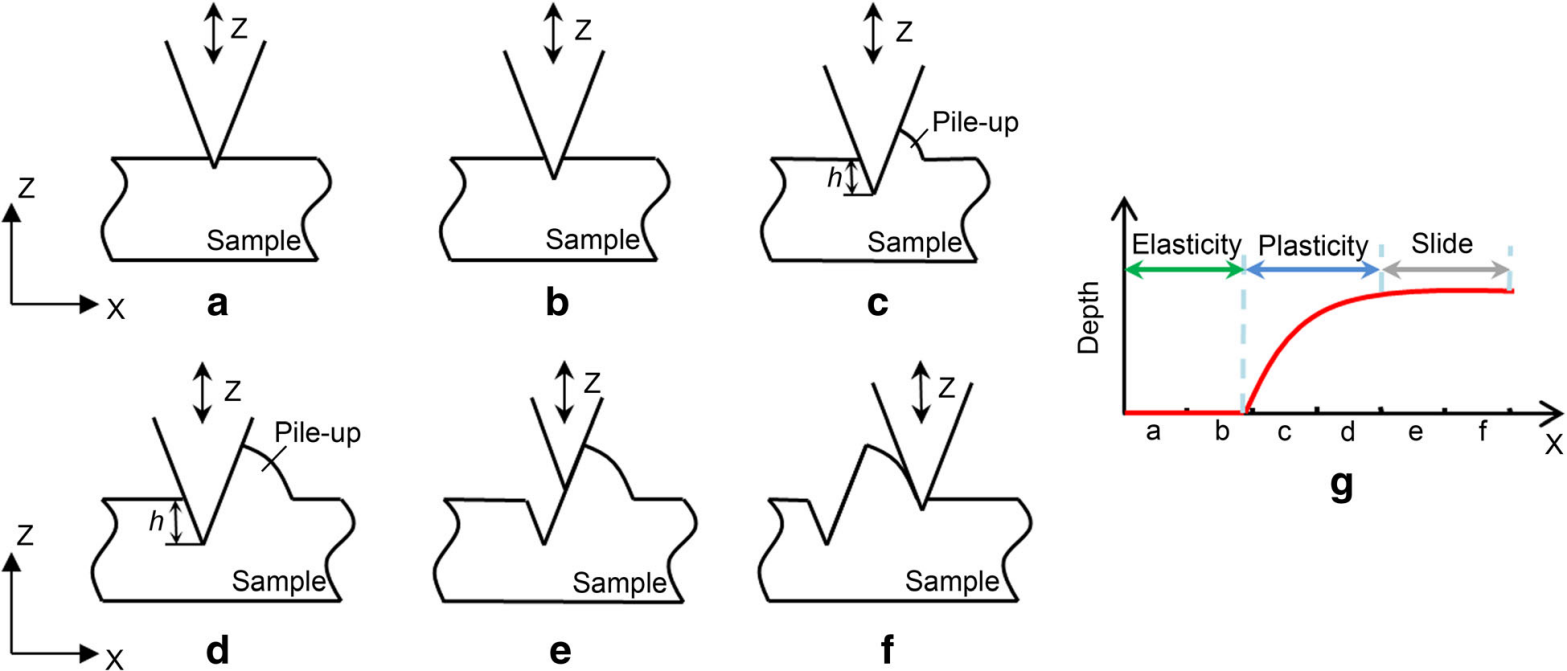
Schematic of pit formation with **a**, **b** elasticity stage, **c**, **d** plasticity stage, **e**, **f** slide stage, and **g** precedence diagram of pit formation

The pit formation process is demonstrated in Fig. 6, including three phases: elastic deformation, plastic deformation, and climbing over the pile-up. According to the above discussion, during the tip scratching across the distance from Fig. 6a, b, the number of press operations is not large enough to break the polymer chains of the PMMA thin film and generate plastic deformation of the sample surface. It has been demonstrated that the oscillating tip penetrates into the polymer sample gradually during the first 40–50 operations [27]. Compared with the indentation process in Ref. [27], pressing with a lateral velocity could generate a distance between two adjacent penetrations. However, the distance between two adjacent penetrations (in the range from 0.52 to 2.33 nm) is much smaller than the radius of the AFM tip (approximately 15 nm). Thus, the situation in this study is similar to the case of the indentation process. Because of a lack of energy accumulation during the initial 30–40 penetrations, no obvious plastic deformation can be found in the machining region. This result indicates that the dominant mechanism of energy dissipation is elastic deformation in the first stage of scratching. Thus, the AFM tip slides in continuous contact with the sample surface during the period of time between Fig. 6a, b. When the number of penetrations conducted by the AFM tip reaches a critical value (40 times in this study), the polymer chains start to break and plastic deformation occurs, as shown in Fig. 6c. At the same time, there would be a normal and shear stress occurring at the interaction surface between the forward face of the AFM tip and the sample material; thus, a pile-up can be generated in front of the forward face of the AFM tip. A strain (Δ) would occur, attributed to the lateral tip motion against the pile-up. This would result in a stress inside the polymer film, which could be released by the propagation of crack [31]. The strain energy release rate *V*_s_ can be described as: [32].

5 $$ {V}_s=E\frac{h}{2}{\left(\frac{\varDelta }{L}\right)}^2 $$

where *E* is the Young’s modulus of the polymer material and *L* is the internal defect length. *h* represents the total penetrating depth to the sample free surface. The surface energy term *W* controls the internal defect process, which is equal to the strain energy release rate through the thermodynamic equilibrium. The surface energy term is dependent on the propagation velocity of the internal defect (*v*_L_), which is given by [33].

6 $$ W={W}_0\left(1+\alpha {v}_L^n\right) $$

where *v*_L_ is equal to d*L*/d*t* and *α* is a constant value related to the sample material. *n* is also a material-related parameter. A tangential force applied on the tip apex could be generated by the elastic energy stored in the polymer substrate, which can be expressed as [32]:

7 $$ {F}_t=\frac{Eah}{2}\frac{\varDelta }{L} $$

where *a* represents the radius of the contact area between the tip and the sample. Because the probe stiffness is much larger than the stiffness of the sample, the material could be removed from the formed hole [31]. However, Mindlin defined a critical value of the force which could result in the tip slipping on the substrate surface [34]. The critical tangential force (*F*_tc_) for the sliding motion could be determined to be a function of the adhesive force and the normal load, expressed as [35–38]:

8 $$ {F}_{tc}=\mu \left(P+3\pi RW+\sqrt{6\pi RW P+{\left(3\pi RW\right)}^2}\right) $$

where *μ* is the coefficient of friction. *P* is the normal, and *R* represents the radius of the AFM tip. When *F*_t_ reaches the critical value *F*_tc_, the AFM tip would slide over the pile-up of the material instead of pushing the material out of the hole produced by the tip. The contact breaks at each cycle, and thus, the slip can thus occur more easily at each cycle in the tapping mode. Although the setting point is not close to 100% of the tip oscillation reduction, a period of contact time could occur during one cycle and the stick may occur during this period of time.

As shown in Fig. 6d, in this study, the depth that the AFM tip penetrated into the sample surface becomes larger because of the decrease of the tip-sample contact area when the AFM tip undergoes a lateral velocity. The height of the pile-up is also increased. This could contribute to balancing the normal load applied by the AFM tip. At the same time, the tangential force applied on the tip apex described in Eq. 7 could also be increased. With an increasing penetration depth, the tangential force could reach the critical value *F*_tc_ given in Eq. 8. The AFM tip starts to slide on the formed pile-up without modifying the material. Because of the characteristics of the tracking sample surface of the AFM system, the AFM tip would rise to climb over the pile-up, as shown in Fig. 6e. After the AFM tip moved over the pile-up, one pit could be achieved and another pit would be fabricated by repeating the above steps. The corresponding deformation mechanism of each stage of pit formation can be found in Fig. 6g.

**Fig. 7:**
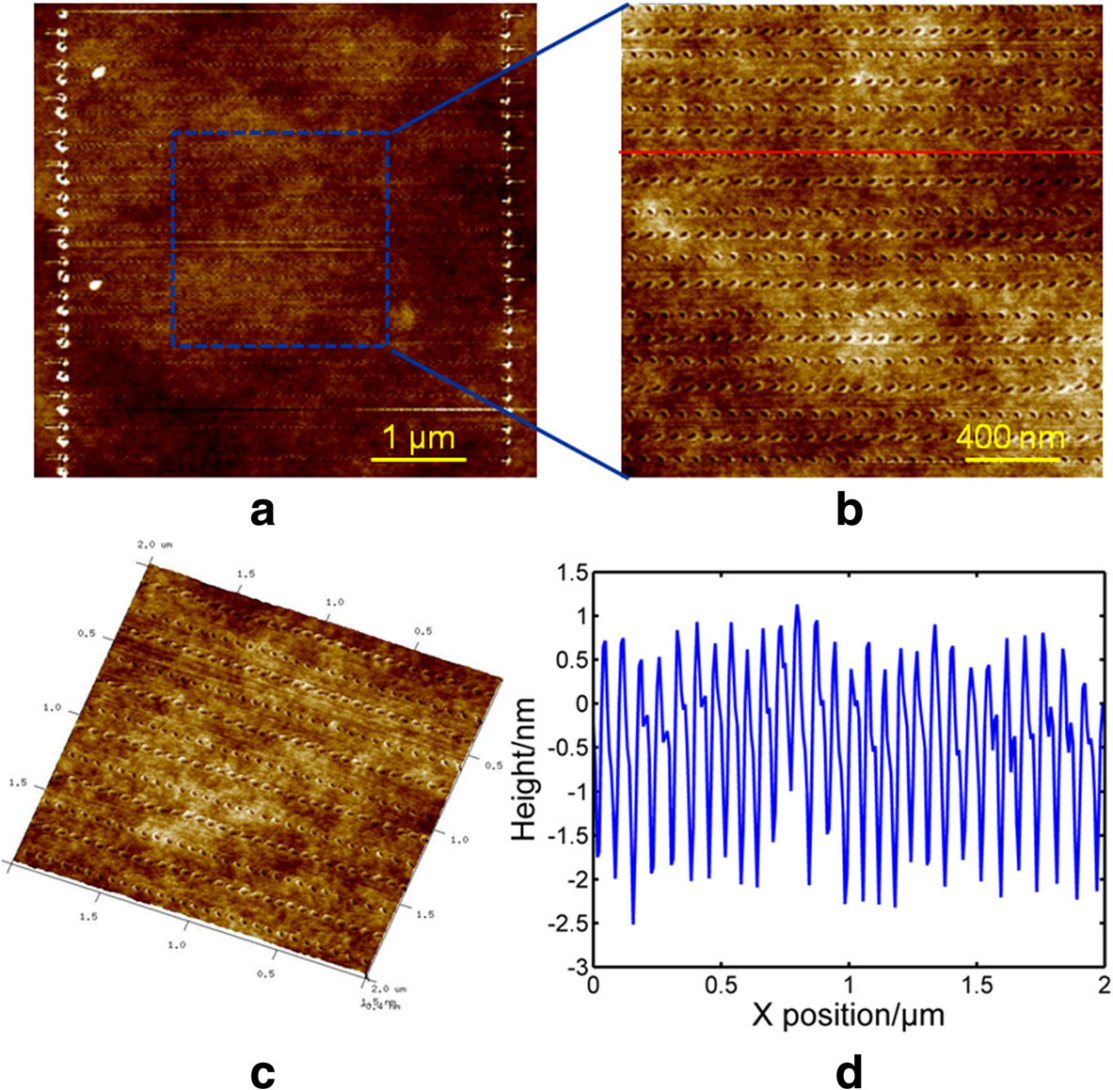
AFM images of an array of pits with a scratching velocity of 400 μm/s, **a** a dimension of 5 μm, **b** a portion of **a** with a dimension of 2 μm, **c** a 3D AFM image of **b**, and **d** a cross-section of pits for the red line in **b**

**Fig. 8:**
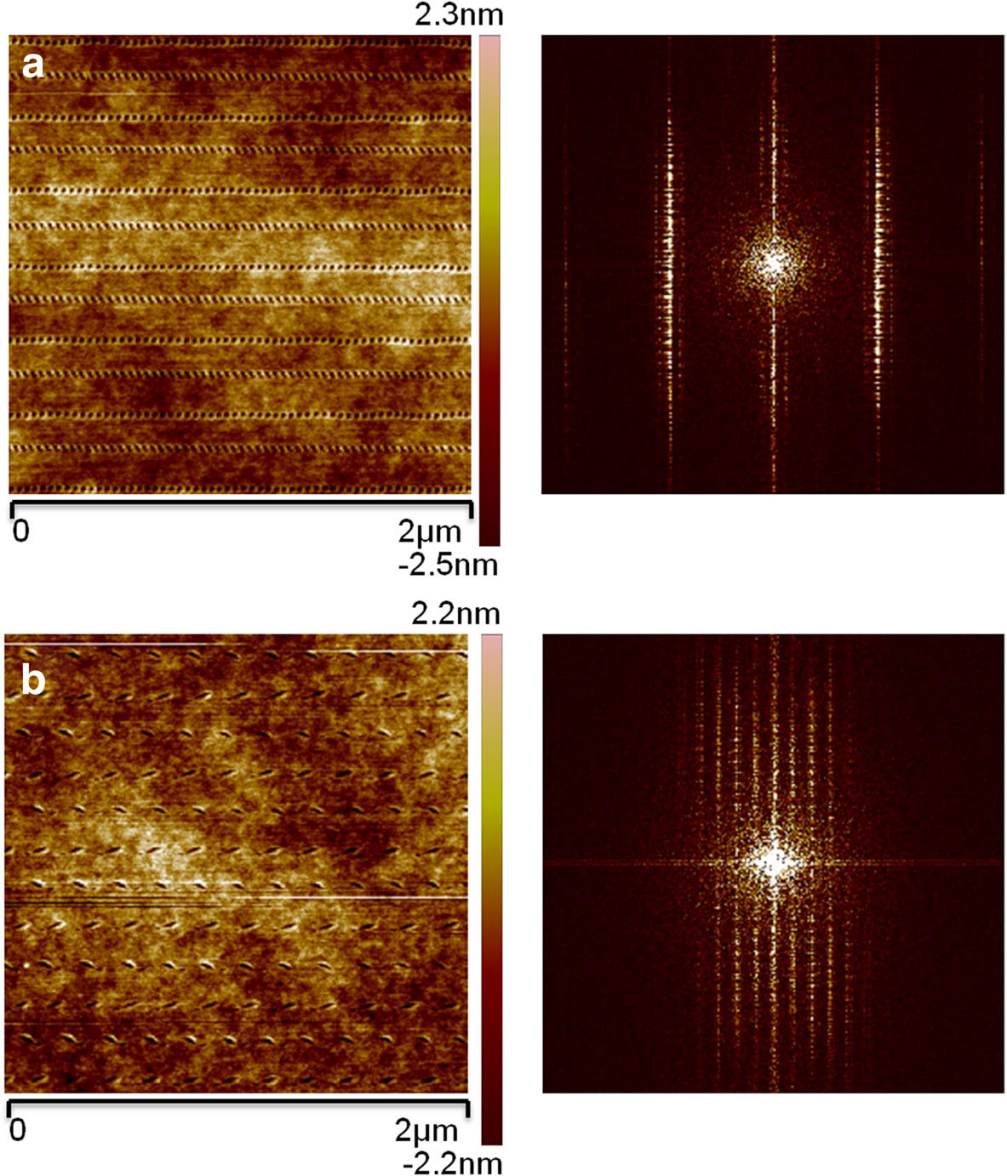
AFM image of pit arrary with a dimension of 2 μm and FFT image of the morphology. The scratching velocities are **a** 200 μm/s and **b** 900 μm/s

According to the previous experimental results, the scratching velocity should be set to larger than 100 μm/s. As shown in Fig. 2b, scratching directions *V*_1_ and *V*_3_ are selected and a feed perpendicular to the scratching direction is conducted to achieve pit arrays with a large dimension of 5 μm. Figure 7a shows the pit arrays obtained with a scratching velocity of 400 μm/s. Figure 7b, c shows the local and 3D AFM images of the machined pits, respectively. Because the scratching velocity slows down to 0 near the transition point of two different scratching directions, the depths of the first and last pits of one horizontal scratching path are much larger than the pits in the middle. One possible reason is explained above. As shown in Fig. 7b, c, the pits in the middle of the scratching path are distributed evenly, which may result from the constant velocity. Moreover, it can be observed from the cross-section of the pits shown in Fig. 7d that the depths of the pits are approximately 2.5 nm. In addition, because of the opposite scratching directions of the adjacent paths, the geometries of the pits in adjacent lines are different. As shown in Fig. 8a, with a scratching velocity of 200 μm/s, the spacing distance between the adjacent pits is relatively small and the geometries of the pits are close to circular. From the fast Fourier transform (FFT) image of the pits, high-density pits can be obtained with a scratching velocity of 200 μm/s. When scratching with a velocity of 900 μm/s, as shown in Fig. 8b, the spacing distance is nearly 100 nm and differences between pits obtained with different scratching directions can be clearly observed. Also from the FFT image of the pits, with a scratching velocity of 900 μm/s, only low-density pits can be achieved.

## Conclusions

To improve the fabrication efficiency with the tip-based DPL method, a scratching velocity that ranges from 0.1 to 1000 μm/s is investigated and demonstrated based on the commercial AFM tapping mode. In the present study, results demonstrate that 100 μm/s is the critical value of the scratching velocity for the formation of pits. Nanogrooves with a pile-up can be obtained with scratching velocities less than the critical value. With scratching velocities greater than 5 μm/s, the machined depths are consistent in all typical directions except the *V*_3_ direction, in which the machined depth becomes much smaller. In contrast, the depth is independent of the scratching direction. Separate pits can be generated with scratching velocities larger than the critical value of 100 μm/s. The total number of fabricated pits can reach nearly 4800–5800 in 1 s, when the scratching velocity is a constant value ranging from 200 to 900 μm/s. According to the stress relaxation modulus theory, the polymer surface is in the condition of a glass state when applying a high-frequency load. The energy applied on the sample surface is not large enough to break PMMA molecular chains during one penetration of the AFM tip. To form one pit, 65 to 80 penetrations are required. For the initial stage of penetration, elastic deformation is the dominant material removal mechanism. When the number of penetrations reaches 40 times, the polymer chains start to break and plastic deformation occurs. With increasing penetration depth, the height of the material accumulated beside the machined pit becomes larger, which will lead to an increase in the tangential force applied on the tip apex. This is the possible reason for the AFM tip sliding over the pile-up, after which one pit is created. Finally, pit arrays with dimensions of 5 μm, spacing distance of 70 nm, and machined depth of 2.5 nm are achieved successfully. FFT images are used to reveal the relationship between the density of pits and the scratching velocity.
